# Urinary Proteome Differences in Canine Diabetes with and without the Presence of Microalbuminuria

**DOI:** 10.3390/ani12060748

**Published:** 2022-03-16

**Authors:** Dagmara Winiarczyk, Mateusz Winiarczyk, Katarzyna Michalak, Stanisław Winiarczyk, Łukasz Adaszek

**Affiliations:** 1 Department of Internal Diseases of Small Animals, University of Life Sciences of Lublin, 20-400 Lublin, Poland; 2 Department of Vitreoretinal Surgery, Medical University of Lublin, 20-059 Lublin, Poland; winiarm86@gmail.com; 3 Department of Epizootiology, University of Life Sciences of Lublin, 20-400 Lublin, Poland; artica@wp.pl (K.M.); genp53@interia.pl (S.W.); ukaszek0@wp.pl (Ł.A.)

**Keywords:** kidney injury, diabetic nephropathy, canine diabetes mellitus, urine markers, proteomic

## Abstract

**Simple Summary:**

Canine diabetes is a serious disease, which can lead to numerous complications. There are limited data on urine proteomics in dogs, and none of the effect of diabetes mellitus on the urine proteome. In this study we aimed to analyze the protein composition of the urine collected from the healthy animals and compare it with two diabetic groups (normoalbuminuric and microalbuminuric). There are significant differences between these three groups, and we believe that the identified proteins hold promise as a potential diagnostic tool, which can be later on used in clinical practice, and for better understanding of the disease.

**Abstract:**

In this study we aimed to analyze the protein composition of the urine collected from the healthy animals and compare it to the two diabetic groups (DM I normoalbuminuric diabetic dogs; DM II diabetic dogs with microalbuminuria). We tried to identify potential urinary proteins which could be up- or downregulated in diabetic patients even before the appearance of microalbuminuria. Methods: After obtaining urine, we performed two-dimensional electrophoresis, followed by Delta2D software analysis, which allowed for selection and identification with MALDI-TOF spectrometry, statistically significant differentially expressed proteins. Our study revealed 286 common protein spots on 2D gels from the diabetic and control group. From these proteins five were positively identified by MALDI-TOF MS. To further evaluate the five differentiating proteins, the Panther program was used to assign them to appropriate biological process. Conclusion: Significant number of identified proteins play a role in intracellular signaling—vesicle formation, bonding, transport through membranes. This may suggest that first signs of kidney diabetic cellular impairment may be seen in the urine composition before any clinical signs occur.

## 1. Introduction

Diabetes mellitus (DM) is a relatively common endocrinopathy occurring mostly in middle-aged and older dogs [1,2]. It can result in numerous complications such as vasculopathy, systemic hypertension, and nephropathy [1]. Due to their short life span, these complications occur infrequently in small animals, but are capable of causing significant disease [3,4]. There is also a paucity of information regarding urine albumin concentration in dogs with spontaneous DM. In a previous study, 20% of diabetic dogs were found to be proteinuric based on a urine protein to creatinine ratio (UPC) > 1 and 46% were hypertensive [5]. Mazzi et al. reported elevated albumin concentration in 55% of the diabetic dogs, with over half of them having concurrent elevation in Urine Protein to Creatinine Ratio (UPC) [6]. In another study the prevalence of microalbuminuria in diabetic patients was in the same level, additionally two of the three dogs with microalbuminuria and normal UPC at initial evaluation developed an elevated UPC during the longitudinal study [4].

Urinary proteins are a promising target for detecting kidney injury. Only a minimal amount of proteins is present in normal urine, due to the mechanical barrier of the glomerulus, and the reabsorption in the proximal tubules. Urinary total protein (UTP) contains proteins originating from filtered plasma, lower urinary tract, and kidney-derived proteins. High urinary protein concentration can be a result of nephron dysfunction, as healthy glomerular filtration barrier excludes proteins larger than 69 kDa, the molecular weight of albumin. Also, it is worth noticing that positively charged proteins pass the glomerular barrier easier than the negatively charged ones. In conditions of disease, glomerular barrier gradually collapses, allowing large amounts of proteins of high, or intermediate weight pass into the ultrafiltrate. Proteins of small molecular weight (>69 kDa) are freely filtered in the glomerulus, but are later reabsorbed by the kidney proximal tubules, therefore both primary and secondary tubular dysfunction can result in proteinuria [7].

Advances in proteomic and metabolomic study have enhanced our ability to identify thousands of proteins and peptides in urine in a single analysis—some of which may serve as new markers. This large-scale study of proteins is termed proteomics, whereas the study of naturally occurring peptides generated by endogenous protease activity is termed peptidomics. Proteomic analysis has become also an important tool in veterinary research [8,9,10,11,12]. The characterization of the canine urinary proteome in healthy dogs has been already investigated [13]. Urinary proteomics and peptidomics add different dimensions to the investigation of underlying biology [14]. Their application in urine has important clinical implications for diabetic kidney disease, given that urine can be collected noninvasively with relative ease and is directly produced by the kidneys. As such, changes in the relative abundance of urinary proteins and peptides may reflect changes in protein expression, deposition, or turnover in the diabetic kidney [15]. Despite well-developed veterinary medicine research concerning diabetic dogs, reports on molecular studies of the urine remains sparse [11,13,14,16,17].

This study aimed to identify proteins in the urine of healthy animals and compare it to the two diabetic groups (normoalbuminuric dogs and dogs with microalbuminuria). We tried to identify potential urinary proteins which could be up- or downregulated in diabetic patients even before the appearance of microalbuminuria.

## 2. Materials and Methods

All the dogs from an outpatient population were consecutively enrolled through the two-year period from 2018–2020 in Innovative Center for Animal Pathology and Therapy Faculty of Veterinary Medicine in Lublin. All samples were obtained during standard veterinary diagnostic procedures; thus, according to Polish law, the approval by Local Commission for Ethics in Animal Experiments was not required since there was no treatment, including medical, invasive diagnostics, or procedures causing psychological or social discomfort for the participants. The experiments were conducted based on the European Union legislation directive 2010/63/EU. The study was carried out in compliance with the ARRIVE guidelines. The dog owners were informed about the methods and purpose of the study and gave their written informed consent. The study was conducted on three groups of dogs: DM I group consisting of 7 normoalbuminuric diabetic dogs (4 male, 3 female, mean age: 7); DM II group consisting of 7 diabetic dogs with microalbuminuria (3 male, 4 female, mean age: 8); H group consist with 7 healthy dogs (4 male, 3 female, mean age: 7).

DM was diagnosed by finding persistent marked hyperglycemia (plasma glucose > 200 mg/dL; 11 mmol/L) and glucosuria in dogs with clinical signs consistent with the disease (polyuria, polydipsia, weight loss).

In addition, owners completed a standardized questionnaire to document changes in water and food consumption, urination frequency, and activity, relative to normal for their pet.

As a study groups, 14 from the total of 30 diabetic mixed-breed dogs presented by the owner were eligible for the inclusion. The inclusion requirements were: (1) a previous diagnosis of diabetes mellitus; (2) insulin treatment given for at least three months; (3) clinically stable diabetes. Exclusion criteria were: proteinuria (Urine Protein to Creatinine Ratio > 0.5), active urinary sediment, pancreatitis, adrenal gland hyperactivity, purulent inflammation of the uterus, and bacterial inflammations of the kidney or urinary bladder. As a control group, 7 healthy dogs of different breeds were included. They were considered healthy based on physical examination, complete blood cell count, plasma biochemistry profile, and urinalysis. The control group was chosen to be age-matched with the study group. Clinical characteristic of the study groups is shown in the Supplementary Table S1.

Clinical examination with blood pressure measurement, routine biochemical and hematological blood tests and urinalysis were performed for each dog. Each blood sample was collected using a closed vacuum system into a test tube containing EDTA and subjected to hematological analysis in an Exigo Vet analyzer (Boule, Spånga, Sweden). The plasma obtained after centrifugation at 3000 rpm for 15 min at 4 °C was analyzed in a BS-130 automatic biochemical analyzer (Mindray, Shenzhen, China). The chemistry panel included alanine transferase, aspartate aminotransferase, total bilirubin, urea, creatinine, alkaline phosphatase, glucose, albumin, total protein, amylase, γ-glutamyltransferase. The concentration of the serum cortisol using enzyme-amplified chemiluminescent assay (Immulite 1000 analyzer) was also determined. For diabetic dogs, glycemic control was estimated using serum fructosamine concentration. Voided midstream urine samples were collected in the morning and each sample was centrifuged on the day of collection at 500× *g* for 10 min at 4 °C. The supernatants were removed and protease inhibitors were added (Protease Inhibitor Cocktail, Sigma, P8340, Spruce Street, Saint Louis, MO, USA.) Urine total proteins and creatinine were determined using commercial kits on an automated chemistry analyzer (Mindray BS-130). The UPC was calculated using the following formula: UPC = urine protein (mg/dL)/urine creatinine (mg/dL). Basic urinalysis with microscopic sediment analysis was performed on fresh urine samples. Urine specific gravity (USG) was measured using a refractometer. Microalbuminuria was measured using a commercially available ELISA test kit (Canine Microalbuminuria Elisa Kit, My Biosource, San Diego, CA, USA). The microalbuminuria test was performed according to the manufacturer’s instructions. Albuminuria was assessed by the urinary albumin-to-creatinine ratio (UACR) in a fresh spot urine sample. The remaining urine was frozen at −80 °C for further analysis.

### 2.1. Proteomic Analysis of Urine

For proteomic analysis, 7 individual urine sample from group I (DM I), 7 individual urine sample from group II (DM II) and 7 individual urine sample from healthy group (H) were collected.

Each urine samples were purified, desalted and concentrated by Amicon Ultra-0.5 3 kDa centrifugal filter units (Merck KGaA, Darmstadt, Germany). Protein concentrations were measured with a micro-volume spectrophotometer (MaestroNano, Maestrogen, Xinzhu, Taiwan), and the urine samples were then prepared and subjected to 2D electrophoresis. After graphical and statistical analysis protein of interest were cut from gel for further identification by mass spectrometry with the MALDI-TOF MS (Matrix-Assisted Laser Desorption Ionization–Time of Flight Mass Spectrometry) technique.

#### 2.1.1. 2D Electrophoresis

Two-dimensional electrophoresis was the first stage of research that results in urine protein separation. Briefly, 200 µg of protein per 17 cm strip was received via a precipitation kit (Ready-Prep™ 2-D Cleanup Kit, Bio-Rad, Hercules, CA, USA). Protein pellets were dissolved in rehydration buffer (ReadyPrep 2-D Rehydration/Sample Buffer 1, Bio-Rad, Hercules, CA, USA), and the resulting solutions were applied to a rehydration plate, covered with 17 cm immobilized pH gradient (IPG) linear strips for isoelectric focusing (ReadyStrip IPG Strips, pH 3–10, Bio-Rad, Hercules, CA, USA) and mineral oil (Bio-Rad, Hercules, CA, USA). After 12 h of rehydration, strips with soaked proteins were transferred to IEF-100 Hoefer apparatus (Hoefer IEF100, Hoefer, Inc., Holliston, MA, USA) to conduct electrophoresis in the first dimension (process conditions: 250 V/30 min; 10,000 V/3 h; 60 kV/hr., with a current limit of 50 μA/strip). Next, strips were equilibrated sequentially in 1,4-dithiothreitol and iodoacetamide urea/TRIS/SDS solutions. Each equilibration step lasted 15 min. Equilibrated strips were then subjected to second dimension of electrophoresis to separated proteins by their molecular masses in 12.5% polyacrylamide gels with the following current parameters: 600 V/30 mA/100 W in an electrophoretic chamber (PROTEAN^®^ II xi, Bio-Rad, Hercules, CA, USA). The obtained gels were subjected to a standard silver-stained procedure with silver nitrate in the presence of formaldehyde. After staining, gels were scanned using the Image Scanner III (GE Healthcare, Chicago, IL, USA), and processed by Delta2D software (version 4.7, DECODON, Greifswald, Germany). Statistical analysis was performed after manually excluding false-positive and false-negative spot. Scanned images were warped by SmartVectors technology in Delta2D software which means that spots of the same protein had the same position across all the gels in the project. Using gel image warping enabled elimination of differences between gel images to align them. Next, warped images were used to create fused image. A fused image responds to the proteome map containing every protein spot obtained in the whole experiment. The expression ratios were generated, and statistics were made over normalized volumes by one-way ANOVA (*p*-value ≤ 0.05) and a post hoc Tukey comparison test. Designated protein spots were cut out of the gels, destained, reduced and alkylated using dithiothreitol and iodoacetamide solutions. Gel pieces containing proteins were subjected to tryptic digestion to obtain peptide fragments. Trypsin digestion occurred in 50 mM ammonium bicarbonate buffer at 37 °C for 12 h (Promega, Trypsin Gold, Mass Spectrometry Grade, Technical Bulletin). Peptides were subsequently eluted from the gel pieces with a water/acetonitrile/TFA solution (*v*:*v* 45:50:5) by triple extraction and received extracts were concentrated in vacuum conditions (Labconco, Kansas City, MO, USA). Obtained peptide pellets were dissolved in 0.1% trifluoroacetic acid and cleaned by C18 Zip-TIP pipette tips according to the manufacturer’s s instructions (Merck Chemicals, Billerica, MA, USA, PR 02358, Technical Note) and prepared to mass spectrometry analysis.

#### 2.1.2. Mass Spectrometry

One µL of prepared peptide solutions were prespotted on an Anchor Chip MALDI plate (Bruker, Bremen, Germany). When the protein samples were dried, their surfaces were covered by 1 μL of α-cyano-4-hydroxycinnamic acid matrix (HCCA, Bruker, Bremen, Germany). Peptide standard solution (Peptide Calibration Standard II, Bruker, Bremen, Germany) was also spotted and covered by matrix on calibration spots. Mass spectra were recorded in active positive reflector mode within the 700–4000 *m*/*z* range using an Ultraflextreme MALDI TOF/TOF (Bruker, Bremen, Germany) spectrometer and the flexControl 3.3 (Bruker, Bremen, Germany) software. Collected spectra were smoothed and baseline corrected. Generated in flexAnalysis 3.0 software (Bruker, Bremen, Germany), peak list for the signal-to-noise ratio of > 3 was transferred to BioTools 3.2 (Bruker, Bremen, Germany), and compared to Mascot 2.2 software (Matrix Science, Boston, MA, USA) using the Swiss-Prot database (www.uniprot.org (accessed on 15 June 2021)) restricted to “mammalia” taxonomy with maximum error 0.3 Da and carbamidomethylation of cysteine as obligatory modification. Results with a Mascot score above 61 were considered statistically significant (*p* ≤ 0.05); otherwise, the fragment ion spectra of chosen peptides were obtained using the LIFT mode and combine in the aim of MALDI TOF/TOF identification.

## 3. Results

Finally, seven normoalbuminuric dogs with diabetes mellitus DM I, seven diabetic dogs with microalbuminuria DM II and seven healthy dogs in the control group were included in proteomic analysis. Diabetic dogs were diagnosed based on clinicopathological variables. They all met the criteria for diabetes mellitus: fasting blood glucose level ≥ 11 mmol/L (200 mg/dL) and glucosuria. Microalbuminuria was defined as an urine albumin to creatinine ratio ≥ 0.1 and ≤ 0.3 [18,19,20]. None of the dogs had signs of chronic kidney disease (systolic blood pressure, serum creatinine and urine protein to creatinine ratio within the physiological range). Clinical characteristics of the study groups are shown in Table 1 and Supplementary Table S1.

**Figure 1:**
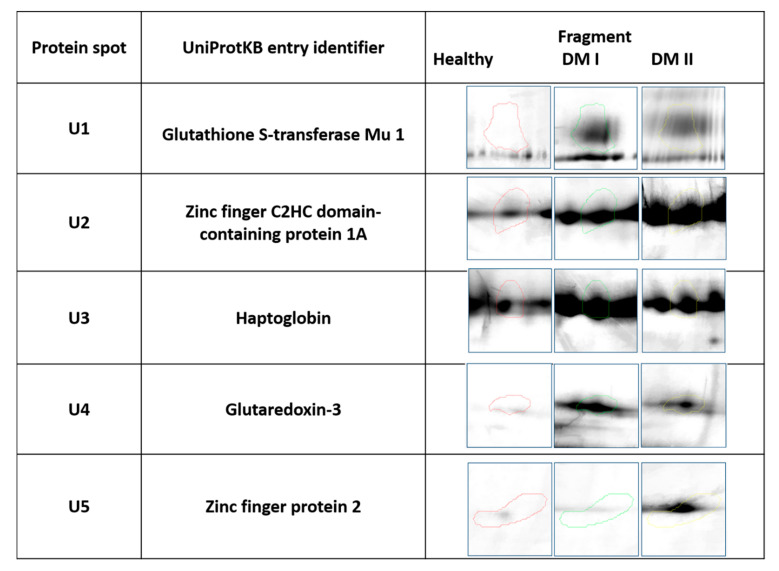
Statistically significant (*p* ≤ 0.05) representatives of 2DE gel spots in the diabetic groups compared to control group as revealed by the Delta2D software. Protein spots form Healthy group are marked in red, protein spot from DM I group are marked in green, Protein spots from DM II group are marked in yellow.

**Figure 2:**
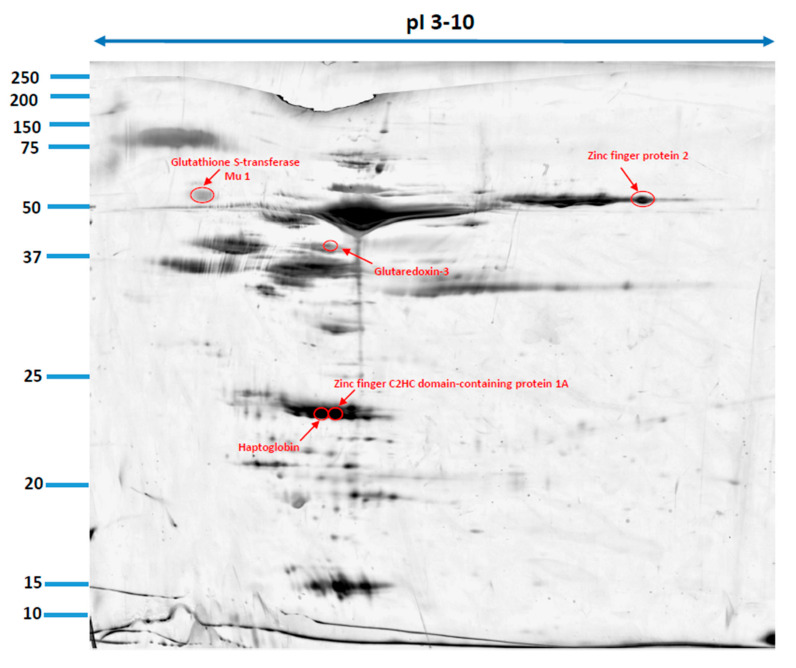
Fused image showing the condensed spot patterns from the experiment. Upregulated proteins are marked in red.

Our study revealed 286 common protein spots on 2D gels from the two diabetic groups and control group. From these proteins 11 have shown statistically significant different expression (*p* ≤ 0.05), and so they were excised from the electrophoretic gel. From these 11 urine proteins, five were positively identified by MALDI-TOF MS. Five proteins which were positively identified by MALDI-TOF were excised from one representative gel of each group. Cut spots were corresponding to each other. Table 2 contains the list of the positively identified proteins, along with their names, genes, and UniProt base accession numbers. With the Delta2D program, one of the five proteins: Zinc Finger Protein 2 was assigned to upregulated just in the group of diabetic dogs with microalbuminuria, whereas in the group of normoalbuminuric diabetic dogs was in the same level compare with the control group. Four of the five identified urine protein (Glutaredoxin-3, Haptoglobin, Zinc finger C2HC domain-containing protein 1A, Glutathione S-transferase Mu 1 were assigned to upregulated in two diabetic groups (Table 2, Figure 1). Figure 1 shows representative two-dimensional electrophoresis gel spots of significantly (*p* ≤ 0.05) differentially expressed proteins in the control group versus the diabetes groups. Figure 2 shows a fused image of the condensed electrophoretic gels from the whole experiment. Ratio–quotient of the group means of relative spot volumes: volume of a given spot in the control group is the denominator of the ratio parameter (Rt > 1.5 overexpression, Rt < 0.67 suppression). To further evaluate the five differentiating proteins, the Panther program (http://www.pantherdb.org (accessed on 15 June 2021)) was used to assign them to the appropriate biological process.

## 4. Discussion

Urine in diabetes is easily accessible source of proteins and was previously investigated in human patients and animal models in many ways, also using proteomic approaches. To the best of our knowledge there is no study regarding proteomic analysis of diabetic canine urine. Our first objective was to find out whether protein profile of canine diabetic urine differ from normoalbuminuric, microalbuminuric and healthy subjects.

The present study demonstrated changes occurring in the urine proteome with diabetes mellitus.

We have identified five differentially expressed urine proteins in diabetic dogs: Glutathione S-transferase Mu 1, Zinc finger C2HC domain-containing protein 1A, Haptoglobin, Glutaredoxin-3, Zinc Finger Protein 2. All these proteins were upregulated in diabetic urine. The sole upregulated protein only in the microalbuminuric dogs was Zinc Finger Protein 2. One could consider linking this protein to the progression of the disease.

Autoimmune process is thought to play a potentially important role in the etiopathology of the canine diabetes and therefore, it was observed in our previous study in diabetic tear film [10], by identified proteins related to inflammation. In this study we investigated urine, and based on the result, we could also confirm this assumption. We observed upregulation of several inflammatory agents, in the urine samples isolated from canine diabetic patients.

Glutathione S-transferase Mu 1 GSTM1 is the major cellular antioxidant that protects against environmental toxicants as well as ROS (oxygen reactive species) mediated cell injury. N-acetylcysteine, a precursor molecule for GSH biosynthesis attenuates diabetes severity in the alloxan induced diabetes model [21] and overexpression of glutamate cysteine ligase, the enzyme involved in the rate limiting step of GSH synthesis, protects pancreatic islets from oxidative stress [22]. GSH detoxifies ROS, reduces peroxides and detoxifies multiple compounds through glutathione-s-transferase (GST) conjugation [21]. In our study we observed the upregulation of Glutathione S-transferase Mu 1 in both diabetic groups. This result suggests that GSTM1 could contribute to the development of diabetic patients. We assume the overexpression of GSTM1 in diabetic urine is a positive, defensive reaction. Further study on canine urine in diabetes mellitus should be conducted, involving larger patient groups to verify the expression of this protein in proteinuric patients.

Haptoglobin (Hp) is a plasma glycoprotein synthesized by hepatocytes and secreted in response to inflammation and infection [23]. The main role of Hp is to bind free haemoglobin (Hb) released during the intravascular destruction of erythrocytes [24], forming the Hp–Hb complex, and by doing so, to prevent oxidative stress on the vasculature. Clinical studies suggested that Hp polymorphism is a risk factor for the development of cardiovascular disease and an independent predictor of cardiovascular outcomes in these patients [25]. There are two common alleles for Hp (1 and 2) and, therefore, three common Hp genotypes: Hp 1-1, Hp 2-1, and Hp 2-2. The antioxidant protection provided by Hp is genotype-dependent; the protein encoded by Hp 1-1 provides superior antioxidant protection compared with that encoded by Hp 2-2. One study shown that diabetic individuals with Hp 2-2 are more likely to develop nephropathy, retinopathy, and cardiovascular disease than those with the Hp 2-1 or Hp 1-1 genotypes [26]. The potential mechanism by which Hp2-2 genotype may cause increased risk of vascular complications is via elevated oxidative stress and/or inflammation. Therefore, decreasing oxidative stress in patients with diabetes using antioxidant supplements may help reduce the risk of cardiovascular events [27]. In our study haptoglobin was identified as an upregulated urine protein in two diabetic groups. We suppose that overexpression in this protein can be associated with intravascular destruction in diabetic dogs. There is a need to determine if the haptoglobin is associated with an increased risk for the development of diabetic nephropathy in a larger group of dogs at various stages of diabetic nephropathy.

Glutaredoxin-3. Glutaredoxins utilize the reducing power of glutathione to maintain and regulate the cellular redox state and redox-dependent signaling pathways, for instance, by catalyzing reversible protein S-glutathionylation. Due to the general importance of these processes, glutaredoxins have been implied in various physiological and disease-related conditions, such as immune defense, cardiac hypertrophy, hypoxia-reoxygenation insult, neurodegeneration and cancer development, progression as well as treatment in humans and animal models [28]. The exact role of Glutaredoxin-3 is still unclear.

Zinc Finger Protein 2. Zinc finger protein ZIC2 is encoded by the ZIC2 gene and can interact with DNA and proteins [29] Gene ontology (GO) annotations related to ZIC2 gene include DNA-binding transcription factor activity and chromatin DNA binding [30]. These proteins are involved in the regulation of multiple steps of RNA metabolism, including mRNA splicing, polyadenylation, transportation, translation and decay. Several zinc finger proteins, are crucial for many aspects of immune regulation by targeting mRNAs for degradation and modulation of signaling pathways [31]. There is lack of information regarding Zinc Finger Protein 2 in diabetes mellitus. It is interesting that in our study we have noticed overexpression of this protein in microalbuminuric urine. Certainly, it is worth to broaden the study and analyze the contribution of Zinc Finger Protein 2 in the pathophysiology of diabetes mellitus.

## 5. Conclusions

In summary, to the best of our knowledge, this study is the first to study the urinary proteome of dogs with diabetes mellitus. It seems that certain specific proteins are expressed in the urine, and those markers can be linked with diabetes mellitus pathology. Our study supports the current view of diabetic complications as a disease where inflammation, oxidative stress, and impaired cellular redox state play a crucial role, making these processes a target for potential future treatment. Due to the limitations of this pilot study, further investigations on canine urine in diabetes mellitus should be conducted, involving larger patient groups to verify their significance in the diagnosis and prognosis of the disease.

## Figures and Tables

**Table 1:** Basic laboratory data of tested dogs.

Variables	Data Format	Healthy (*n* = 7)	DM I (*n* = 7)	DM II (*n* = 7)
CBC				
WBC (White Blood Cells) [×10^9^/L]	Mean (SD)	7.49 (1.33)	9.42 (3.10)	8.25 (3.4)
RBC (Red Blood Cells) [×10^12^/L]	Mean (SD)	7.46 (0.63)	6.61 (0.67)	7.23 (0.34)
PCV (Packed Cell Volume) [%]	Mean (SD)	46.65 (3.91)	41.27 (5.61)	45.32 (4.67)
HGB (Hemoglobin) [g/dL]	Mean (SD)	13.75 (1.54)	15.09 (1.73)	14.42 (2.21)
PLT (Platelet Count) [×10^9^/L]	Mean (SD)	355.92 (72.82)	445.54 (140.08)	387.54 (132.21)
Biochemical parameters				
Fasting blood glucose [mmol/L]	Mean (SD)	<11	18.54 (3.64)	17.43 (4.23)
Serum Fructosamine [µmol/L]	Mean (SD	234.23 (21.34)	328.2 (32.54)	331.23 (45.32)
Serum Urea [mmol/L]	Mean (SD)	5.99 (1.31)	6.44 (2.97)	6.21 (43)
Serum Creatinine [µmol/L]	Mean (SD)	63.65 (13.24)	54 (14.35)	51 (12.32)
Total Protein [g/dL]	Median (IQR) *	6.1(0.9)	6.8 (0.30)	6.6 (0.3)
Urinary parameters				
UPC	Mean (range)	0.02 (0.003–0.08)	0.15 (0.04–0.20)	0.17 (0.10–0.20)
UAC	Mean (range)	0.002 (0.0005–0.01)	0.7(0.04–0.10)	0.28 (0.16–0.30)
USG	Mean	1.030	1.025	1.030

* interquartile range; UPC: urine protein to creatinine ratio; UAC: urine albumin to creatinine ratio; USG: urine specific gravity; SD: Standard Deviation.

**Table 2:** Significantly (*p* ≤ 0.05) differentially expressed proteins in diabetic dogs identified by MALDI-TOF MS.

ID	Protein	Accesion Number (UniProtKB)	Species	Score	Match	MW (Da) *	pI **	Seq. Cov (%)	Rt *** DMI/H	Rt DMII/H
**U1**	Glutathione S-transferase Mu 1	P10649	*M. musculus*	92	8	26,067	7.71	25	2.965	3.053
**U2**	Zinc finger C2HC domain-containing protein 1A	A4FUE7	*B. taurus*	78	6	35,718	9.92	13	2.552	1.995
**U3**	Haptoglobin	P19006	*C. lupus familiaris*	73	9	36,890	5.72	24	2.729	1.981
**U4**	Glutaredoxin-3	Q58DA7	*B. taurus*	63	4	37,559	5.56	14	3.365	2.053
**U5**	Zinc finger protein 2	P08043	*M. musculus*	64	7	54,154	9.10	21	1.001	3.424

Listed molecular weights and pI values correspond to the MASCOT Search Result, carbamidomethylation of cysteine was a global modification; * Monoisotopic mass; ** Calculated pI, *** Rt (Ratio) quotient of the group means of relative spot volumes; volume of a given spot in control group is the denominator of the ratio parameter; MW: molecular weight; pI: isoelectric point.

## Data Availability

The data presented in this study are available on repository ftp://MSV000088968@massive.ucsd.edu (accessed on 15 June 2021).

## Supplementary Materials

The following supporting information can be downloaded at: https://www.mdpi.com/article/10.3390/ani12060748/s1, Table S1: Clinical characteristic of the study subjects.
