# Supplementary material for: Urinary Proteome Differences in Canine Diabetes with and without the Presence of Microalbuminuria
Source: Animals (Basel). 2022 Mar 16;12(6):748. doi: 10.3390/ani12060748 (PMC8944454; doi:10.3390/ani12060748)
Supplement: Supplementary file 1 [file animals-12-00748-s001.zip › Table S1.pdf]

Table S1. Clinical and biochemical parameters for the healthy, DM I, and DM II subjects.

	Unit	Healthy (n=7)	DM I (n=7)	DM II (n=7)
Gender (M/F)	None	4M, 3F	4M, 3F	3M,4F
Age	Years	7 [5-9]	7 [6-9]	8 [5-10]
BCS	None	5	6 [3-7]	6 [3-7]
Duration of diabetes mellitus	month	0	23 [7-48]	35 [9-48]
Systolic Blood Pressure	mmHg	123 [110-148]	154 [120-178]	159 [134-169]
Serum Fructosamine	µmol/L	234 [210-252]	328 [317-340]	331 [320-369]
Urine PCR	-	0.02 [0.003-0.08]	0.15 [0.04-0.20]	0.17 [0.10-0.20]
Urine ACR	-	0.002 [0.0005-0.01]	0.7[0.04-0.10]	0.28 [0.16-0.30]

BCS: body condition score; Urine PCR: urine protein to creatinine ratio; Urine ACR: albumin urine to creatinine ration
